# Usefulness of Self-Assessment of Gastrointestinal Symptoms: Web-Based Study in Anhui, China

**DOI:** 10.2196/42101

**Published:** 2023-08-30

**Authors:** Xiaoqin Guan, Qun Xue, Huan Ma, Guocheng Li, Xiuze Xu, Kexin Zhang, Mengsha Tang, Rong Liu, Debin Wang, Xingrong Shen

**Affiliations:** 1 School of Health Service Management Anhui Medical University Hefei, Anhui China; 2 Center for Operational Health Service Research Anhui Medical University Hefei, Anhui China

**Keywords:** gastrointestinal symptoms, health care seeking, symptoms reporting, web-based self-assessment, China

## Abstract

**Background:**

Gastrointestinal symptoms (GISs) are caused by a combination of biopsychosocial factors and are highly prevalent worldwide. Given their complex nature, coupled with ineffective communication of diagnoses by physicians, patients with intimate GISs often feel stigmatized. This, in turn, can inhibit their ability to express their thoughts and feelings adequately, leading them to over- or underreport their symptoms. Moreover, selective service-seeking for and reporting of GISs have a direct bearing on the stage of disease at presentation and, consequently, on the overall prognosis.

**Objective:**

This study aimed to investigate the usefulness of a web-based self-assessment of GISs as a supplementary means to cope with potential over- or underreporting during routine consultations.

**Methods:**

GIS data were collected using a novel web-based self-assessment tool (n=475) and from nonparticipative observation of doctor-patient consultations (n=447) and household surveys (n=10,552) in Anhui, China. Data analysis focused primarily on the description of the composition of respondents and the occurrence rates of GISs by sociodemographics, and by symptom solicitation methods and settings. Chi-square power tests were used when necessary to compare differences in the occurrence rates between relevant groups. The level of significance for the 2-sided test was set at α<.05.

**Results:**

The average occurrence rates of both upper and lower GISs derived from the web-based self-assessment were higher than those from the observation (upper GISs: n=661, 20.9% vs n=382, 14.2%; *P*<.001; lower GISs: n=342, 12.9% vs n=250, 10.8%; *P*=.02). The differences in 6 of the 9 upper GISs and 3 of the 11 lower GISs studied were tested with statistical significance (*P*<.05); moreover, a higher frequency rate was recorded for symptoms with statistical significance via self-assessment than via observation. For upper GISs, the self-assessed versus observed differences ranged from 17.1% for bloating to 100% for bad mood after a meal, while for lower GISs, the differences ranged from −50.5% for hematochezia or melena to 100% for uncontrollable stool. Stomachache, regurgitation, and dysphagia had higher occurrence rates among participants of the self-assessment group than those of the household survey group (20% vs 12.7%, 14% vs 11%, and 3% vs 2.3%, respectively), while the opposite was observed for constipation (5% vs 10.9%), hematochezia or melena (4% vs 5%), and anorexia (4% vs 5.2%). All differences noted in the self-assessed occurrence rates of specific, persistent GISs between sociodemographic groups were tested for nonsignificance (*P*>.05), while the occurrence rates of any of the 6 persistent GISs among respondents aged 51-60 years was statistically higher than that among other age groups (*P*=.03).

**Conclusions:**

The web-based self-assessment tool piloted in this study is useful and acceptable for soliciting more comprehensive GISs, especially symptoms with concerns about stigmatization, privacy, and shame. Further studies are needed to integrate the web-based self-assessment with routine consultations and to evaluate its efficacy.

## Introduction

Gastrointestinal diseases (GIDs) or functional gastrointestinal disorders (FGIDs) are highly prevalent worldwide, leading to substantial distress and health care burden [1]. In the United States, GIDs affect 60-70 million citizens, with health care expenditures accounting for US $135.9 billion dollars annually [2]. There have been reports of more than 40.7 million ambulatory visits for gastrointestinal symptoms (GISs), and 54.4 million visits with a primary diagnosis of a GID each year [3]. The major symptoms of common GIDs include recurrent abdominal pain and bloating, heartburn, dyspepsia, nausea and vomiting, diarrhea, and constipation. These symptoms are common to a broad range of organic pathology, including gastrointestinal cancer, inflammatory bowel disease, coeliac disease, peptic ulcer, and motility disorders such as gastroparesis. However, it is well recognized that for a substantial proportion of patients with GISs, investigation reveals no underlying structural abnormality that can explain these symptoms, which are then often referred to as FGIDs, such as irritable bowel syndrome (IBS), functional dyspepsia (FD), or functional constipation. Although not completely understood, FGIDs account for at least a third of referrals to gastroenterology clinics, and nearly half of the general population have functional disorders [4-7]. In addition, FGIDs feature a long course and repeated symptoms and thus cause serious and enduring physical, psychological as well as sociological distress [8-10]. GIDs or FGIDs in China are also most prevalent. According to a study carried out in medical care settings, the prevalence of FD and IBS was about 18.9%-36.8% and 5%-6%, respectively [11]. Another study conducted in Guangdong province revealed that the prevalence of FGIDs among urban residents was 46.4% (49.8% for female participants and 43.1% for male participants), and the highest rate of consultation was for FD (60.7%), followed by IBS (53.3%) [12].

GISs are the result of a combination of biopsychosocial factors. For example, a substantial link was observed between the severity of IBS and its comorbid psychiatric disorders, particularly depression and anxiety [13,14]. Given their complex nature, coupled with ineffective communication of diagnoses by physicians, patients with intimate GISs often feel stigmatized. This stigma can, in turn, inhibit the patient’s desire to seek professional help and ability to express their thoughts and feelings adequately to their physicians, causing them to over- or underreport their symptoms [15,16]. Such selective service-seeking for and reporting of GISs may have a direct bearing on the stage of disease at presentation and, consequently, on the overall prognosis [17]. Bearing these factors in mind, we developed a novel web-based self-assessment tool for patients to preinput their GISs in a hope to offset potential under- or overreporting during routine consultations. This study explores the usefulness of such a tool by comparing the GISs derived from the self-assessment, and from direct observation of doctor-patient consultations, and a household survey.

## Methods

### Participant Recruitment

The study used data from 2 separate sources, that is, the baseline investigation of a randomized controlled trial (ISRCTN41836539) and a household survey of common symptoms and health conditions that was conducted in Anhui, China. The baseline investigation involved patient self-assessment and nonparticipative observation of the consultation process in the outpatient departments of 24 county hospitals, drawn throughout Anhui province via cluster randomization. It included 8 hospitals each in the northern, central, and southern regions. Participant recruitment in any given outpatient department lasted for 2 weeks; all incoming patients to the department within the 2 weeks were checked for eligibility, and all eligible patients were invited to participate. The inclusion criteria comprised male and female participants who were (1) 18-70 years old; (2) seeking medical care for gastrointestinal conditions; and (3) able and willing, after informed consent, to participate. Of the 2 weeks of recruitment, the first week was spent in nonparticipative observation, and the second week was dedicated to self-assessment. If the hospital opened more than 1 consultation room a day, the observation covered all the rooms in turn at 1 room a day. In addition, all the doctors involved were told that the study was aimed at comparing the differences in patients’ conditions and their consultation process at different levels and settings leading to a potential user-friendly decision support system without giving any details of forthcoming self-assessment.

The household survey participants were also selected via stratified randomization proceeded through (1) dividing the whole Anhui province into north, middle, and south regions; (2) randomly selecting 4 counties from each of the regions; and (3) randomly drawing 5 townships from each of the counties selected as the study site; (4) randomly selecting 60 residents with any GISs from the communities selected. The inclusion criteria were the same as above.

### Data Sources and Collection

Of the 2 data sources mentioned above, the self-assessment took place at the outpatient department of the randomized controlled trial site hospitals and before the clinical consultation (when patients were waiting to see their doctors). It adopted a web-based, dual-model, and structured questionnaire. “Dual-model” here means that the questionnaire was presented in both text and voice formats to enable the illiterate patients to perform the self-assessment. The observation happened at the participating outpatient department proceeded as: a researcher seated in the select consultation room, observed the patient-doctor encounters, and recorded the process according to a preset checklist. The household survey took place at the household of the respondents and used a structured questionnaire administered face-to-face by trained medical graduate students. All the observations, self-assessments, and household surveys happened in the summertime from 2019 to 2021.

Data extracted from the above sources for this study were mainly social demographics (eg, age, sex, and education) and current and persistent GISs (eg, stomachache, abdominal pain, nausea, and vomiting). Here, current GISs refers to the then-presenting symptoms when the consultation or survey took place, and persistent GISs defines symptoms that had been occurring repeatedly from time to time for over 6 months. The questions used for soliciting data about both the current and persistent GISs were self-designed based on a literature review and rounds of field tests. The Cronbach α of the current and persistent symptoms were estimated as .803 and .703, respectively. The observation and self-assessment included both the current and persistent GISs, while the household survey included only the persistent GISs.

### Statistical Analysis

The completed checklists and questionnaires were double-entered into a database using EPI DATA (version 3.1; The EpiData Association) and then analyzed using SPSS (version 20; IBM Corporation) and Microsoft Excel (version 2016; Microsoft Corp). Cases with missing data were excluded. The data analysis focused primarily on the description of the occurrence rates of GISs derived from different methods, settings, and sociodemographic groups. Chi-square power tests were used to compare differences in the occurrence rates of GISs between relevant groups. The level of significance for the 2-sided test was set at α<.05.

The data analysis comprised a description of 5 categories of variables. First, the compositions of participants by sociodemographics (sex, age, and education level) and data collection methods (observation, web-based self-assessment, and household survey) were described. Second, the occurrence rates of current GISs from the self-assessment were compared with that from the observation. Third, the occurrence rates of persistent GISs from the self-assessment were compared with that from the household survey. Fourth, the occurrence rates of current GISs were estimated from the self-assessment by participant sociodemographic groups. Finally, the occurrence rates of persistent GISs were computed from the self-assessment and household survey by sociodemographics.

### Ethical Considerations

The study protocol was reviewed and approved by the Biomedical Ethics Committee of Anhui Medical University (reference 20210648) prior to commencement of the study. Participation of residents was voluntary, and written informed consent was obtained from all participants. Participant data were anonymous or deidentified.

## Results

### Social Demographics of Included Participants

In all, 947 patients in the outpatient departments of hospitals and 11,500 residents from the household survey were approached. A total of 922 (self-assessment = 475, observation = 447) and 10,552 completed questionnaires were collected by these participants, respectively. As shown in Table 1, the self-assessment group consisted of 228 (48%) male participants and 247 (52%) female participants; the mean age was 50.1 (SD 15.2) years. Nearly half of them were illiterate or had elementary school or lower education. The observation group consisted of 214 (47.9%) male participants and 233 (52.1%) female participants; the mean age was 50.5 (SD 14.7) years. Respondents in the household survey group consisted of 4248 (40.3%) male participants and 6304 (59.7%) female participants; the mean age was 57.8 (SD 10.0) years. There existed statistically significant differences between the 3 groups in terms of sex, age, and education with the outpatient subjects being younger, more educated, and more even in sex.

**Table 1:** Sociodemographics of patients recruited in outpatient departments and households.

Characteristics		Observation	Self-assessment	Household survey	*P* value	
**Sex, n (%)**						<.001
	Male	214 (47.9)	228 (48.0)	4248 (40.3)		
	Female	233 (52.1)	247 (52.0)	6304 (59.7)		
**Age (years),** **n** **(%)**						<.001
	≤40	68 (15)	110 (23.2)	294 (2.8)		
	41-50	86 (19)	132 (27.8)	2485 (23.6)		
	51-60	91 (20)	121 (25.5)	3164 (30.0)		
	>60	73 (16)	112 (23.6)	4549 (43.1)		
**Education (years),** **n** **(%)**						<.001
	0	62 (14)	90 (19)	2871 (27.2)		
	1-5	102 (22.8)	144 (30.3)	2982 (28.3)		
	6-8	92 (21)	134 (28.2)	2795 (26.5)		
	>8	62 (14)	107 (22.5)	1811 (17.2)		

### Current GISs From Self-Assessment and Observation

As shown in Table 2, the most frequent upper GISs observed during the outpatient consultation was stomachache (n=90, 30.1%), followed by bloating (n=89, 29.8%), eructation (n=61, 20%), regurgitation (n=56, 19%), and nausea and vomiting (n=28, 9%). Although the most frequently observed lower GISs was abdominal pain (n=75, 39%), followed by unformed stool (n=36, 19%), hematochezia or melena (n=30, 15%), diarrhea (n=29, 15%), and constipation (n=27, 14%). Putting together, both the average occurrence rates of upper and lower GISs derived from the self-assessment were higher than that from the observation (n=661, 20.9% vs n=382, 14.2%; *P*<.001 and n=342, 12.9% vs n=250, 10.8%; *P*=.02, respectively). The differences in 6 of the 9 upper GISs and 3 of the 11 lower GISs were tested with statistical significance (*P*<.05), and all these symptoms witnessed higher self-assessed than observed frequency rates. For upper GISs, the self-assessment versus observation difference ranged from 17.1% for bloating to 100% for bad mood after a meal, while for the lower GISs, the difference ranged from −50.5% for hematochezia or melena to 100% for uncontrollable stool. The mean length of the self-assessment and patient-doctor encounter was estimated as 9.60 (SD 3.59) minutes and 3.29 (SD 2.57) minutes, respectively.

**Table 2:** Current gastrointestinal symptoms derived from self-assessment and observation.

Current gastrointestinal symptoms		Self-assessment (a)		Observation (b)	Difference^a^		*P* value
**Upper** **gastrointestinal** **symptoms, n (%)**							
	Stomachache	149 (42.4)	90 (30.1)		29.1	.001	
	Bloating	126 (35.9)	89 (29.8)		17.1	.10	
	Eructation	100 (28.5)	61 (20)		28.4	.02	
	Regurgitation	92 (26)	56 (19)		28.5	.02	
	Heartburn	51 (14)	18 (6)		58.6	<.001	
	Anorexia	49 (14)	17 (6)		59.2	<.001	
	Nausea and vomiting	46 (13)	28 (9)		28.6	.13	
	Dysphagia	40 (11)	23 (8)		32.5	.11	
	Bad mood after a meal	8 (2)	0 (0)		100	.009	
	Total symptoms	661 (20.9)	382 (14.2)		32.1	<.001	
**Lower** **gastrointestinal** **symptoms, n (%)**							
	Abdominal pain	75 (34)	75 (39)		−14.5	.30	
	Unformed stool	56 (25)	36 (19)		26.4	.10	
	Abdominal distension	50 (23)	23 (12)		47.3	.004	
	Diarrhea	36 (16)	29 (15)		7.7	.72	
	Increased stool	29 (13)	16 (8)		36.8	.11	
	Constipation	28 (13)	27 (14)		−10.4	.69	
	Hematochezia or melena	25 (10)	30 (15)		−50.5	.21	
	Borborygmus	21 (9)	11 (6)		40	.15	
	Stool with mucus	10 (4)	2 (1)		77	.03	
	Anal pain	8 (4)	1 (0.5)		85.6	.04	
	Uncontrollable stool	4 (2)	0 (0)		100	.13	
	Total symptoms	342 (12.9)	250 (10.8)		12.1	.02	

^a^(a − b) / a × 100%.

### Persistent GISs Derived From Self-Assessment and Household Survey

Table 3 compares the occurrence rates of persistent GISs the self-assessment (at the outpatient departments of the site hospitals) and household survey. The overall occurrence rates of the 6 persistent GISs studied were estimated as 51.2% (n=243) from the self-assessment and 47.1% (n=4968) from the household survey. Stomachache, regurgitation, and dysphagia had higher occurrence rates among participants of the self-assessment group than those of the household survey group (n=96, 20% vs n=1336, 12.7%, n=67, 14% vs n=1165, 11%, and n=14, 3% vs n=246, 2.3%, respectively), while the opposite was observed for constipation (n=24, 5% vs n=1149, 10.9%), hematochezia or melena (n=21, 4% vs n=527, 5%), and anorexia (n=21, 4% vs n=545, 5.2%). Of the differences in the occurrence rates of all 6 GISs studied between the 2 groups, 3 were tested with statistical significance. The biggest difference in occurrence rate between the 2 groups was noted for constipation (115.6%), followed by stomachache (37.4%) and regurgitation (21.8%).

**Table 3:** Persistent gastrointestinal symptoms derived from self-assessment and household survey.

Gastrointestinal symptoms	Self-assessment, n (%) (a)	Household survey, n (%) (b)	Difference^a^	*P* value
Stomachache	96 (20)	1336 (12.7)	37.4	<.001
Regurgitation	67 (14)	1165 (11.0)	21.8	.04
Constipation	24 (5)	1149 (10.9)	−115.6	<.001
Hematochezia or melena	21 (4)	527 (5.0)	−12.9	.57
Anorexia	21 (4)	545 (5.2)	−16.7	.47
Dysphagia	14 (3)	246 (2.3)	21.0	.39
Total	243 (51.2)	4968 (47.1)	8.0	<.001

^a^(a − b) / a × 100%.

### Current GISs From Self-Assessment by Sociodemographics

Table 4 provides estimated rates of current GISs from self-assessment by sociodemographics. Most of the differences in the rates of specific GISs between sex, age, and education groups were tested without statistical significance except for bloating, unformed stool, increased stool, and diarrhea. More specifically, bloating presented a decreasing trend with education (*P*=.03), while respondents with over 8 years of education witnessed a higher occurrence rate of unformed stool (*P*=.001) and increased stool (*P*=.048). In addition, female participants were more likely to self-report diarrhea than male participants (*P*=.03).

**Table 4:** Current gastrointestinal symptoms from self-assessment by sociodemographics.

Characteristics			Sex				*P* value		Age (years)										*P* value			Education (years)												*P* value	
			Male		Female				≤40		41-50	51-60		>60							0				1-5		6-8			>8					
**Upper gastrointestinal symptoms, n (%)**																																			
	Stomachache	76 (42)		73 (38)		.38		29 (34)		43 (44)			43 (43)		34 (37)		.49			35 (46)			45 (40)					46 (41)			23 (32)				.05
	Bloating	62 (34)		64 (34)		.75		27 (32)		38 (39)			30 (30)		31 (34)		.84			28 (37)			38 (34)					43 (38)			17 (24)				.03
	Eructation	46 (25)		54 (28)		.65		20 (24)		35 (36)			27 (27)		18 (20)		.20			17 (22)			24 (21)					36 (32)			23 (32)				.20
	Regurgitation	41 (22)		51 (27)		.46		25 (29)		28 (29)			23 (23)		16 (18)		.40			18 (24)			19 (17)					30 (27)			25 (35)				.14
	Heartburn	20 (11)		31 (16)		.18		8 (9)		21 (22)			10 (10)		12 (13)		.12			7 (9)			14 (13)					19 (17)			11 (15)				.45
	Anorexia	21 (11)		28 (15)		.45		15 (18)		11 (11)			11 (11)		12 (13)		.56			11 (14)			15 (13)					9 (8)			14 (19)				.37
	Nausea and vomiting	21 (11)		25 (13)		.74		14 (16)		12 (12)			11 (11)		9 (10)		.66			11 (14)			8 (7)					13 (12)			14 (19)				.18
	Dysphagia	21 (11)		19 (10)		.55		9 (11)		9 (9)			9 (9)		13 (14)		.56			8 (11)			15 (13)					12 (11)			5 (7)				.43
	Bad mood after a meal	5 (3)		3 (2)		.41		2 (2)		2 (2)			3 (3)		1 (1)		.82			1 (1)			2 (2)					3 (3)			2 (3)				.91
**Lower gastrointestinal symptoms, n (%)**																																			
	Abdominal pain	36 (35)		39 (32)		1.00		19 (33)		23 (34)			18 (35)			15 (33)	.80			10 (30)				22 (37)		21 (32)			22 (34)			.34			
	Abdominal distension	26 (25)		24 (20)		.55		15 (26)		19 (28)			10 (19)			6 (13)	.07			4 (12)				15 (25)		16 (24)			15 (23)			.16			
	Unformed stool	28 (27)		28 (23)		.75		19 (33)		20 (29)			12 (23)			5 (11)	.01			3 (9)				14 (23)		16 (24)			23 (35)			.001			
	Increased stool	17 (17)		12 (10)		.24		12 (21)		8 (12)			5 (10)			4 (9)	.09			3 (9)				5 (8)		9 (14)			12 (18)			.048			
	Borborygmus	13 (13)		8 (7)		.19		5 (9)		10 (15)			3 (6)			3 (7)	.17			3 (9)				2 (3)		9 (14)			7 (11)			.10			
	Hematochezia or melena	12 (12)		13 (11)		1.00		7 (12)		5 (7)			7 (13)			6 (13)	.82			4 (12)				5 (8)		9 (14)			7 (11)			.58			
	Diarrhea	11 (11)		25 (20)		.03		12 (21)		12 (18)			7 (13)			5 (11)	.23			3 (9)				10 (17)		10 (15)			13 (20)			.13			
	Constipation	11 (11)		17 (14)		.34		4 (7)		9 (13)			4 (8)			11 (24)	.12			7 (21)				11 (18)		5 (8)			5 (8)			.43			
	Others	9 (9)		13 (11)		.50		5 (9)		4 (6)			4 (8)			9 (20)	.40			6 (18)				3 (5)		8 (12)			5 (8)			.32			

### Persistent GISs From Self-Assessment by Sociodemographics

Table 5 presents the 6 most common persistent GISs by methods of symptom collection (self-assessment vs household survey) and sociodemographics of respondents. For self-assessment, the occurrence rates of specific persistent GISs between sociodemographic groups were tested for nonsignificance (*P*>.05). Yet, the overall occurrence rates of the 6 GISs studied had statistically significant variations (*P*=.03) between age groups, with higher reports among respondents aged 51-60 years. For household surveys, most of the intrasubgroup differences in the rates of GISs were tested with statistical significance (*P*<.05). The younger and the less educated, the greater the chances for them to report the persistent GISs. Female participants were more likely to report constipation but less likely to report hematochezia or melena.

**Table 5:** Persistent gastrointestinal symptoms by sociodemographics and symptom collection method.

Characteristics				Total		Regurgitation		Dysphagia	Stomachache		Anorexia	Constipation		Hematochezia or melena
**Self-assessment**														
	**Sex, n (%)**													
		Male	97 (43)		36 (16)		8 (4)		41 (18)	8 (4)		9 (4)	10 (4)	
		Female	104 (42.1)		31 (13)		6 (2)		55 (22)	13 (5)		15 (6)	11 (4)	
		*P* value	.92		.31		.49		.25	.35		.29	.97	
	**Age (years), n (%)**													
		<40	42 (38)		15 (14)		3 (3)		19 (17)	8 (7)		5 (5)	3 (3)	
		41-50	51 (39)		21 (16)		1 (1)		26 (20)	4 (3)		6 (5)	7 (5)	
		51-60	65 (54)		23 (19)		4 (3)		33 (27)	4 (3)		6 (5)	7 (6)	
		>60	43 (38)		8 (7)		6 (5)		18 (16)	5 (4)		7 (6)	4 (4)	
		*P* value	.03		.06		.21		.14	.38		.93	.63	
	**Education (years), n (%)**													
		0	47 (52)		10 (11)		4 (4)		26 (29)	5 (6)		6 (7)	2 (2)	
		1-5	62 (43)		21 (15)		3 (2)		30 (21)	5 (3)		9 (6)	5 (3)	
		6-8	54 (40)		22 (16)		6 (4)		23 (17)	3 (2)		3 (2)	9 (7)	
		>8	38 (45)		14 (13)		1 (1)		17 (16)	8 (7)		6 (6)	5 (5)	
		*P* value	.12		.71		.30		.10	.22		.36	.39	
**Household survey**														
	**Sex, n (%)**													
		Male	1328 (31.3)		479 (11.3)		111 (2.6)		526 (12.4)	214 (5.0)		335 (7.9)	252 (5.9)	
		Female	2019 (32.0)		686 (10.9)		135 (2.1)		810 (12.8)	331 (5.3)		814 (12.9)	275 (4.4)	
		*P* value	.41		.53		.12		.48	.63		<.001	<.001	
	**Age (years), n (%)**													
		<40	132 (44.9)		45 (15)		7 (2)		52 (18)	19 (6)		55 (19)	29 (10)	
		41-50	851 (34.2)		268 (10.8)		44 (2)		352 (14.2)	124 (5.0)		304 (12.2)	144 (5.8)	
		51-60	943 (29.8)		326 (10.3)		85 (3)		409 (12.9)	129 (4.1)		315 (10.0)	156 (4.9)	
		>60	1398 (30.7)		519 (11.4)		107 (2.4)		514 (11.3)	266 (5.8)		466 (10.2)	197 (4.3)	
		*P* value	<.001		.046		.16		<.001	.004		<.001	<.001	
	**Education (years), n (%)**													
		0	1004 (35.0)		404 (14.1)		87 (3)		434 (15.1)	217 (7.6)		285 (9.9)	136 (4.7)	
		1-5	1010 (33.9)		358 (12.0)		80 (3)		420 (14.1)	166 (5.6)		324 (10.9)	167 (5.6)	
		6-8	863 (30.9)		265 (9.5)		51 (2)		322 (11.5)	109 (3.9)		337 (12.1)	140 (5.0)	
		>8	442 (24.4)		128 (7.1)		25 (1)		151 (8.3)	48 (3)		192 (10.6)	82 (5)	
		*P* value	<.001		<.001		<.001		<.001	<.001		.08	.32	

## Discussion

### Principal Results

This study unveiled useful information on the usefulness of the web-based self-assessment of GISs. First, the study revealed substantial differences between the occurrence rates of current GISs derived from the self-assessment and that from the observation of the patient-doctor interactions. Both the average occurrence rates of upper and lower GISs derived from the web-based self-assessment were higher than that derived from the observation. Second, the study uncovered subtle differences in specific symptoms obtained from the self-assessment versus the observation. For upper GISs, the self-assessed versus observed differences ranged from 17.1% for bloating to 100% for bad mood after a meal, while for lower GISs, the differences ranged from −50.5% for hematochezia or melena to 100% for uncontrollable stool. Third, the study identified interesting differences between the occurrence rates of persistent GISs from the self-assessment and the household survey. Stomachache, regurgitation, and dysphagia had higher occurrence rates among participants of the self-assessment group than those of the household survey group, while the opposite was observed for constipation, hematochezia or melena, and anorexia. Fourth, the study documented almost no statistically significant variations in the occurrence rates of persistent GISs from the self-assessment across sociodemographic groups.

### Comparison With Prior Work

To the best of our knowledge, this is the first study that tries to use web-based self-assessment of GISs to offset potential under- or overreporting during routine consultation in China. Given the high prevalence of GISs and the complex factors (including social, system, and policy contexts) affecting symptom reporting, each of the findings mentioned above has important implications. The differences between the occurrence rates of GISs from the self-assessment versus observation may be attributed to several reasons. In China, a doctor working at county-level hospitals generally needs to see some 40 to 70 patients in the morning, and the doctor-patient consultation time is very short [18]. In this study, it was only 3.29 (SD 2.57) minutes on average. Faced with a long queue of patients outside the consultation room, doctors may have to interrupt the patient's reporting from time to time and thus inhibit the patients from fully expressing their feelings and thoughts. Meanwhile, the patients’ perceived time pressure may have made them to be selective in reporting their symptoms, presumably choosing the most important and distressing symptoms. By comparison, the self-assessment had no time limit and lasted 9.60 (SD 3.59) minutes on average. It used a structured and systematic approach and asked for all potential symptoms. In addition, the self-assessment was performed, while the patients were waiting for their consultation and used only their idle time which otherwise often causes worries [19]. These suggest that the web-based self-assessment piloted in this study is not only acceptable and practical but also beneficial in collecting more comprehensive information and easing worries about waiting for consultation.

The study identified differences between the occurrence rates of specific symptoms from the self-assessment versus observation. Although the overall frequency of symptoms reported in the self-assessment was substantially higher than that in doctor-patient consultation, the occurrence of part of the symptoms, for example, dysphagia and diarrhea, was very close (*P*=.11 and *P*=.72, respectively). This may partly be explained by variations in perceived disgrace, shamefulness, and stigmatization. Some symptoms like anal pain and stool with mucus may make the patient feel shameful or disgraceful to talk in front of other people, while the self-assessment was performed in an unobtrusive and “person-to-machine” context, giving the patient enough ease and privacy to report. Other symptoms like bloating and abdominal distension are often labeled as “functional” symptoms, and thus lead to various stigmatization due to, for example, societal taboos around discussions of bowel functioning, and historically viewing these disorders as psychosomatic [20]. There are reports that half of all patients with FGIDs do not even inform their family members and friends about the diagnosis because of fear of being misunderstood or not believed [21]. There are also publications that organic disorders have traditionally been given greater credence, so doctors are more ready to devote their attention to “organic” or “sicker” patients [22,23].

The differences between the occurrence rates of persistent GIS from the self-assessment versus the household survey may due mainly to perceived significance of the symptoms under concern and interactions between symptoms. On one hand, the participants in the self-assessment were in fact outpatients with GISs presenting to the site hospitals, while the respondents of the household survey were general community residents. On the other hand, for patients, more symptoms may mean severe and more complex diseases and thus merits higher-level professional health care. Considering these, the higher overall occurrence rate of persistent GISs among the self-assessment participants versus the household survey respondents may due largely to severe GISs among the former than the latter group. Following this logic, the specific persistent GISs should all show higher occurrence rates from the self-assessment than the household survey. Yet, 3 of the 6 persistent GISs studied demonstrated the opposite trend. This may be explained by (1) inaccurate estimation due to a very small number (less than 24) of cases from the self-assessment; (2) varied perceptions of GISs (eg, patient may view constipation, hematochezia, and anorexia as less important/painful than the others). In addition, the study findings suggest that current or acute GISs may be more important drivers for seeking professional care, since 48.8% (n=232) of the outpatients (self-assessment participants) had not reported any persistent GISs; yet all of them had reported multiple current GISs.

The differences between the occurrence rates of persistent GISs from the self-assessment and household survey by sociodemographic groups are consistent with published studies. The Rome Foundation global survey showed that 49% of women reported at least 1 FGID compared with 37% of men [4]. This may partly explain why the self-assessment had recruited more female patients than male patients. Similarly, the decreasing or increasing tendency of occurrence rates of GISs among age or education groups may be linked, to some extent, to health-related conditions and awareness. There is evidence that younger adults are more health conscious than older adults and have better health conditions and access to health services [24]. There are also indications that those more educated are younger and that education level is negatively associated with inattention to treatment, suggesting educated people may be more concerned about their health probably because of better knowledge of the consequences of not receiving the appropriate treatment [25].

### Strengths and Limitations

The study has both strengths and limitations. It is advantageous since it tested the usefulness and feasibility of a novel web-based self-assessment tool for systematically soliciting GISs when the patients were waiting for their outpatient consultation. In addition, it compared the differences in GISs derived using different venues, at different settings, and from different perspectives; these differences have important implications for clinical practice, patient care, and future research. For example, introducing web-based self-assessment prior to patient-doctor consultation may result in more complete GISs, less workload for doctors, and greater satisfaction among patients. The study also has some drawbacks. First, it used only univariate descriptive analysis of the differences between groups, which did not exclude interactions between variables studied. Second, the study involved nonparticipative observation, which may have induced some extent of influence on the practice behaviors. For example, when being observed, the doctors may practice more rigorously according to perceived standards and ask for more details from their patients. Third, readers may raise the concern about the potential effects of prior self-assessment on the patient’s later consultation. Such effects merit careful evaluation in similar studies; yet they should not affect our findings as presented since the consultations of patients being observed were different from those who had performed the self-assessment. Fourth, the study was implemented in limited months rather than a whole year, and readers are cautioned about potential seasonal variations.

### Conclusions and Suggestions

The web-based self-assessment tool piloted in this study is useful and acceptable. It helps in soliciting more comprehensive GISs, especially symptoms with concerns about stigmatization, privacy, shame, and so forth. Yet, further studies are needed to integrate the web-based self-assessment with routine consultations and evaluate its efficacy.

In addition to further studies on the above self-assessment, the study findings also have important implications both for clinical practice and community education. For clinical practice, physicians need to acknowledge the distress caused by functional disorders, give even credence to both organic and functional symptoms, and let the patient report his/her symptoms without interruption first and then use the order of symptoms reported to optimize the forthcoming consultation by not only paying adequate attention to address the first few symptoms but also enquiring symptoms that are overlooked by patients. For community education, patients need to be properly educated about the importance of complete symptoms (especially often underreported GISs) reporting, techniques talking about sensitive symptoms, and understanding acute versus persistent GISs.

## Abbreviations

FD: functional dyspepsia
FGID: functional gastrointestinal disorder
GID: gastrointestinal disease
GIS: gastrointestinal symptom
IBS: irritable bowel syndrome

## Data Availability

The data sets generated and analyzed during this study are not publicly available but are available from the corresponding author on reasonable request.
